# Pharmacokinetic and pharmacogenomic predictors of hepatotoxicity in the HIRIF trial for drug-susceptible tuberculosis

**DOI:** 10.1093/cid/ciag141

**Published:** 2026-03-06

**Authors:** Eunsol Yang, Pieter Van Brantegem, Charles A. Peloquin, Meredith B. Brooks, Julia M. Coit, Dante Vargas Vásquez, Epifanio Sánchez Garavito, Roger I. Calderón, Judith Jiménez, Karen Tintaya, Dalicxa Garcia, Elna Osso, Elizabeth A. Mackay, Gareth Platt, Leonid Lecca, Radojka M. Savic, Geraint R. Davies, Carole D. Mitnick, Gustavo E. Velásquez

**Affiliations:** 1 Department of Bioengineering and Therapeutic Sciences, University of California, San Francisco, San Francisco, California, USA; 2 College of Pharmacy and Emerging Pathogens Institute, University of Florida, Gainesville, Florida, USA; 3 Department of Global Health, Boston University School of Public Health, Boston, Massachusetts, USA; 4 Department of Global Health and Social Medicine, Harvard Medical School, Boston, Massachusetts, USA; 5 Hospital Nacional Hipólito Unanue, Lima, Peru; 6 Hospital Nacional Sergio Bernales, Lima, Peru; 7 Advanced Research and Health, Lima, Peru; 8 Facultad de Medicina Humana, Universidad de San Martín de Porres, Lima, Peru; 9 Socios En Salud Sucursal Peru, Lima, Peru; 10 Department of Clinical Infection, Microbiology, and Immunology, University of Liverpool, Liverpool, United Kingdom; 11 UCSF Center for Tuberculosis, Institute for Global Health Sciences, University of California, San Francisco, San Francisco, California, USA; 12 Division of Global Health Equity, Brigham and Women’s Hospital, Boston, Massachusetts, USA; 13 Partners In Health, Boston, Massachusetts, USA; 14 Division of HIV, Infectious Diseases, and Global Medicine, University of California, San Francisco, San Francisco, California, USA

**Keywords:** Drug-susceptible tuberculosis, hepatotoxicity, rifampin, isoniazid, pyrazinamide, *NAT2* acetylator

## Abstract

**Background:**

Hepatotoxicity is frequent in the standard-of-care regimen for drug-susceptible tuberculosis, yet the association of each companion drug with hepatotoxicity has not been fully characterized. We examined the association between hepatotoxicity and the pharmacokinetics of rifampin and its companion drugs in standard therapy, as well as N-acetyltransferase 2 (*NAT2*) genotype.

**Methods:**

We evaluated HIRIF trial participants who were randomized to receive rifampin 10, 15, or 20 mg/kg/day during the intensive phase of tuberculosis treatment. We fitted Cox proportional hazards models to identify risk factors for grade 2 or higher (grade 2+) alanine transaminase (ALT) or aspartate transaminase (AST) elevation, and considered pharmacokinetic exposure of each antituberculosis drug and *NAT2* genotype as potential covariates.

**Results:**

Among 168 participants with pharmacokinetic data, neither rifampin dose nor exposure were associated with the risk of grade 2+ ALT or AST elevation. Higher pyrazinamide exposure (hazard ratio [HR] 1.85 for every 50 mg*h/L AUC_0–6h_ increase), higher isoniazid exposure (HR 1.40 for every 5 mg*h/L AUC_0–6h_ increase), and among a subset with known *NAT2* genotype, slow *NAT2* acetylator status (HR 9.32 relative to fast, n=90) were associated with grade 2+ ALT or AST elevation in univariable analysis. In multivariable analysis, only pyrazinamide exposure was associated with hepatotoxicity.

**Conclusions:**

While higher pyrazinamide and isoniazid exposures and slow *NAT2* acetylator status were each associated with increased hepatotoxicity, only pyrazinamide exposure was associated when considering pharmacokinetic variables together. Rifampin exposure was not associated with hepatotoxicity, supporting further evaluation of doses up to 20 mg/kg/day in a larger trial.

## INTRODUCTION

Tuberculosis (TB) remains the leading infectious cause of death [1]. Despite the availability of highly efficacious treatment, morbidity and mortality among TB patients remain substantial, partly due to adverse events induced by antituberculosis drugs, which can lead to treatment discontinuation or interruption, increasing the risk of treatment failure, relapse, and drug resistance [1,2].

The 6-month regimen comprising isoniazid, rifampin, pyrazinamide, and ethambutol has been the standard therapy for drug-susceptible TB (DS-TB) since the 1980s [3,4]. Hepatotoxicity is one of the most frequent and severe adverse events occurring with this regimen, with reported incidence ranging from 2% to 28% [5]. While asymptomatic transaminase elevations are common during treatment, hepatotoxicity can be fatal if not recognized early and managed appropriately through timely treatment interruption [5]. Given that treatment interruption significantly erodes treatment efficacy [6], preventing drug-induced hepatotoxicity is critical to ensuring treatment success and patient safety during TB therapy.

Among the first-line antituberculosis drugs for DS-TB, isoniazid, rifampin, and pyrazinamide are known to have hepatotoxic properties, but their individual contributions to hepatotoxicity remain unclear due to their use in combination [7]. N-acetyltransferase 2 (*NAT2*) genotype, which primarily affects the extent of isoniazid metabolism and its resulting exposure, has also been linked to anti-TB drug-induced hepatotoxicity [8–10]. Furthermore, with growing interest in shortening the treatment duration for DS-TB by increasing rifampin doses [11–14], isolating the hepatotoxic impact of higher rifampin exposure is essential to ensuring its safe clinical use. To date, however, the quantitative contribution of each component drug to hepatotoxicity within standard therapy for DS-TB, particularly in the context of increased rifampin doses, has not been well characterized.

The Phase 2B dose-ranging trial, HIRIF (NCT01408914) [14], previously showed the potential of higher rifampin doses to shorten standard therapy without added toxicity. In the present analysis, we used data from the HIRIF trial to evaluate the association between hepatotoxicity and the pharmacokinetics (PK) of rifampin and its companion drugs in the standard of care regimen for DS-TB. We also explored the potential contribution of *NAT2* genotype to hepatotoxicity.

## METHODS

### Patient population

We evaluated adults with newly diagnosed, smear-positive, pulmonary DS-TB enrolled in the Phase 2B HIRIF trial (NCT01408914). Participants were randomized to receive rifampin 10, 15, or 20 mg/kg/day during the intensive phase of treatment in a 1:1:1 ratio. The detailed trial design and participant characteristics have been previously described [14,15].

### Pharmacokinetic data

The following previously reported PK parameters of isoniazid, rifampin, pyrazinamide, and ethambutol were utilized in this study: area under the plasma concentration-time curve (AUC) from 0 to 6 h post-dose (AUC_0–6h_) and maximum plasma concentration (C_max_) [16]. For participants whose isoniazid AUC_0–6h_ was not calculable, AUC from 0 to last measurable time point (AUC_last_) values were used as imputed substitutes. The detailed design and methodology for the PK analysis have been previously described [16].

### Pharmacogenomic data

Genotyping data for *NAT2* was available in this study, as previously described [17]. *NAT2* genotype was categorized into slow, intermediate, and fast acetylator status based on the single *NAT2* tag nucleotide polymorphism (rs1495741).

### Outcome

We defined hepatotoxicity as the time to grade 2 or higher alanine aminotransferase (ALT) or aspartate aminotransferase (AST) elevation during the intensive phase and up to 4 weeks thereafter (i.e., a total of 12 weeks), based on the primary safety endpoint used in the HIRIF trial [14].

### Statistical analysis

All data processing, statistical analyses, and visualizations were conducted using R version 4.2.3 (R Foundation for Statistical Computing, Vienna, Austria), and a p-value < 0.05 was considered statistically significant. We compared the PK parameters for each drug between participants with and without the hepatotoxicity outcome using an independent two-sample *t*-test. We explored the effects of randomization or baseline characteristics on the PK parameters using an independent two-sample *t*-test, one-way analysis of variance (ANOVA), or linear regression, as applicable.

We performed Cox proportional hazards analysis to identify risk factors for hepatotoxicity, with PK parameters and *NAT2* genotype as predictors of interest. We generated Kaplan-Meier estimates stratified by each drug’s AUC_0–6h_ dichotomized at the population median and by *NAT2* genotype. In the univariable Cox analysis, we tested PK parameters and *NAT2* genotype as potential risk factors, and assessed other demographic and baseline clinical factors as potential confounders. We evaluated the proportional hazards assumption for all variables. For the multivariable Cox analysis, we included only PK parameters due to limited availability of *NAT2* genotype and performed complete case analysis without imputation. We selected candidate risk factors for the final multivariable model using a stepwise procedure, testing linear relationships through forward inclusion (p < 0.05) and backward exclusion (p < 0.01).

We developed longitudinal models for ALT and AST using a nonlinear mixed-effects modeling approach with NONMEM version 7.5.1 (ICON plc, Gaithersburg, Maryland) to investigate whether the velocity (slope) of elevation was associated with exposure to individual drugs. We censored longitudinal trajectories of ALT and AST at the first grade 2 or higher elevation event, respectively. We explored structural models using linear or turnover models, including a linear or maximum effect (E_max_) relationship with time. We described the two subpopulations representing low and high elevations of ALT and AST using a probabilistic mixture model that assigned individuals to each subpopulation based on their observed data, enabling the estimation of distinct slope profiles within the population [18]. First, we compared the PK parameters for each drug between the mixture model-assigned subpopulations of ALT and AST elevation using an independent two-sample *t*-test. Second, we evaluated the influence of drug exposure on the slopes of ALT and AST elevation using linear regression and a stepwise approach with forward selection (p < 0.05) and backward elimination (p < 0.01). We performed model selection based on numerical and visual assessments, including significance-based selection on log-likelihood testing, the precision of parameter estimates, goodness-of-fit plots, and visual predictive checks.

## RESULTS

### Participant characteristics

Among the 180 adults randomized in the HIRIF trial, 168 participants with available PK data and 90 participants with available *NAT2* genotype regardless of PK data availability were included in the present analysis. Baseline characteristics, including weight and transaminase levels, and observed PK parameters for isoniazid, pyrazinamide, and ethambutol were similar across randomized rifampin dose groups (Table 1). In the 168 participants with available PK data, pyrazinamide exposure was positively correlated with isoniazid, rifampin, and ethambutol exposures, and ethambutol exposure was positively correlated with rifampin exposure (Supplementary Figure 1). Of the 90 participants with available *NAT2* genotype, 32 (17.8%) were classified as slow, 42 (23.3%) as intermediate, and 16 (8.9%) as fast acetylator status. Isoniazid exposures increased across fast, intermediate, and slow acetylator status (Supplementary Figure 2).

### Risk Factors for Grade 2 or Higher ALT or AST Elevation

Grade 2 or higher ALT or AST elevation was observed in 39 (23%) of the 168 participants with available PK data, with a median time to event of 27 (range: 13–68) days, and in 24 (27%) of the 90 participants with available *NAT2* genotype, with a median time to event of 27 (range: 12–56) days. Time to grade 2 or higher ALT or AST elevation did not show any apparent trend across randomized rifampin dose groups (Figure 1). Pyrazinamide and isoniazid exposures were significantly higher in participants with grade 2 or higher ALT or AST elevation compared with those without elevation, while rifampin and ethambutol exposures were similar regardless of elevation (Figure 2).

In univariable Cox analysis, higher exposures to pyrazinamide and isoniazid, and slow *NAT2* acetylator status among the subset with known *NAT2* genotype, were associated with an increased hazard of grade 2 or higher ALT or AST elevation: hazard ratio [HR] 1.85 (95% CI, 1.23–2.81) for every 50 h∙mg /L increase in pyrazinamide AUC_0–6h_; HR 1.40 (95% CI, 1.04–1.87) for every 5 h∙mg/L increase in isoniazid AUC_0–6h_; HR 9.32 (95% CI, 1.22–70.92) for slow relative to fast *NAT2* acetylator status (Table 2, Figure 3). Neither rifampin dose nor exposure, nor ethambutol exposure, or other demographic or clinical factors were associated with the hazard of grade 2 or higher ALT or AST elevation (Table 2, Supplementary Table 1). A sensitivity analysis of the Cox analyses described above adjusted for randomized rifampin dose yielded the same independent risk factors as the unadjusted analyses (Supplementary Table 2).

In the final Cox model, only higher pyrazinamide exposure remained significantly associated with an increased hazard of grade 2 or higher ALT or AST elevation (Table 2). All other candidate risk factors did not meet statistical criteria for inclusion in the model after adjusting for pyrazinamide exposure. A sensitivity analysis in the subset of 84 participants with both available PK data and *NAT2* genotype showed that higher isoniazid exposure and slow *NAT2* acetylator status retained a nonsignificant trend towards increased hazard of grade 2 or higher ALT or AST elevation (Supplementary Table 3).

### Longitudinal ALT/AST analysis

A total of 891 ALT and 958 AST values from 168 participants were included in the longitudinal analysis (Supplementary Figure 3). The longitudinal ALT and AST profiles were well described by linear models, combined with a two-subpopulation mixture model representing low and high elevations (Supplementary Figure 4, Supplementary Figure 5, Supplementary Table 4). While exposures to pyrazinamide and isoniazid were significantly higher in the model-assigned high ALT and AST elevation groups compared to the low elevation groups, exposure to rifampin was similar across the model-assigned elevation groups (Supplementary Figure 6), consistent with the findings from survival analysis. The model-predicted slopes of ALT and AST elevation were significantly associated with exposures to isoniazid, pyrazinamide, and ethambutol, but not rifampin (Figure 4). Drug exposure parameters, however, did not meet the prespecified statistical criteria for inclusion and thus were not included in the final longitudinal model (Supplementary Table 4).

## DISCUSSION

We found that rifampin exposures achieved with doses up to 20 mg/kg/day were not associated with hepatotoxicity, further supporting the topline results of the HIRIF trial, which observed no increased risk of hepatic adverse events with higher rifampin doses [14]. Although our dataset was moderately sized, we did not observe any clear trends in the hazard of hepatotoxicity with higher rifampin exposure in either univariable or full multivariable models, indicating no apparent association. The current standard dose of rifampin (10 mg/kg) is widely considered subtherapeutic and has been associated with unfavorable treatment outcomes [19,20]. Accumulating evidence from both preclinical and clinical studies has shown the potential of higher rifampin doses to enhance therapeutic efficacy without a significant increase in safety concerns [21–27], making it a promising strategy to shorten treatment duration of the standard regimen for DS-TB. While prior studies primarily linked rifampin dose levels to safety outcomes, our study directly evaluated the association between continuous rifampin PK, which more precisely reflect drug exposure in the body, and hepatotoxicity risk. Our results provide robust rationale that higher rifampin doses up to 20 mg/kg pose little concern for hepatotoxicity compared to the current standard, thereby supporting its clinical use from a safety perspective.

Higher pyrazinamide exposure was the strongest predictor of hepatotoxicity in this study. Our findings align with the largest pyrazinamide PK-safety analysis to date. Data from the Study 31/ACTG A5349 (S31/A3549) Phase 3 trial showed that increased pyrazinamide exposure in the 6-month standard regimen was associated with a higher risk of ALT or AST ≥5 times the upper limit of the normal range [28]. While the precise mechanisms underlying pyrazinamide-induced hepatotoxicity are not fully understood, increased pyrazinamide metabolites have been reported to be highly correlated with the extent of hepatotoxicity [29], underscoring the importance of optimizing pyrazinamide exposure.

Since treatment interruption can often resolve hepatotoxicity but may also result in suboptimal drug exposure and potential treatment failure and drug resistance [5], it is crucial to proactively minimize pyrazinamide overexposure. Therapeutic drug monitoring (TDM) of pyrazinamide could help optimize exposure, however, the current capacity for TDM in high-burden clinical settings is limited. Pending increased capacity, flat dosing of pyrazinamide could be a feasible strategy to mitigate the risk of hepatotoxicity in the standard regimen for DS-TB. The S31/A3549 pyrazinamide analysis highlighted that flat dosing of pyrazinamide 1000 mg ensures a higher proportion of participants remain within the therapeutic window compared with weight-banded dosing, particularly by reducing the risk of high pyrazinamide exposure—and toxicity—in higher weight bands [28].

We found that higher isoniazid exposure and slow *NAT2* acetylator status were significant predictors of hepatotoxicity in univariable analysis. *NAT2* genotype was not included in the multivariable analysis due to limited availability, and in the final model, the contribution of higher isoniazid exposure to hepatotoxicity was eclipsed by the outsize contribution of pyrazinamide exposure. Our findings indicate that optimizing isoniazid exposure may still be important for minimizing hepatotoxicity in the standard DS-TB regimen. Isoniazid is predominantly metabolized by the highly polymorphic *NAT2* enzyme, and isoniazid-induced hepatotoxicity is known to be primarily attributed to its toxic metabolites, the extent of which depends on *NAT2* enzyme activity [5]. Accordingly, *NAT2* genotype associated with slow acetylation has been reported to increase the likelihood of hepatotoxicity due to the accumulation of toxic metabolites [7–10]. This supports the notion that slow *NAT2* acetylator status, identified as a risk factor in our analysis, is an underlying determinant of isoniazid-related hepatotoxicity risk.

*NAT2* genotype-guided isoniazid dose adjustment has been proposed to reduce the risk of isoniazid-induced hepatotoxicity while improving clinical efficacy [30–35]. Furthermore, decreased acetyl-isoniazid levels have been reported to be associated with hepatotoxicity, suggesting the potential for acetyl-isoniazid levels as a biomarker for isoniazid-related hepatotoxicity risk [36,37]. Both genotype- and PK-guided approaches could be beneficial for optimizing isoniazid doses, thereby minimizing the risk of hepatotoxicity in the standard therapy for DS-TB, although practical challenges remain.

In our longitudinal analysis of ALT and AST trajectories, we found that the model-predicted slopes of ALT and AST elevation were significantly and independently associated with exposures to isoniazid, pyrazinamide, and ethambutol, but not rifampin. However, these PK parameter associations did not meet prespecified statistical criteria for inclusion in the final longitudinal model. These findings align with the results from the survival analysis, further suggesting that pyrazinamide and isoniazid, rather than rifampin, are contributors to hepatotoxicity.

Only results for ethambutol were divergent between the two analyses. Although ethambutol is generally not considered a major contributor to hepatotoxicity in standard therapy for DS-TB [7], we noted a significant correlation between its AUC_0–6h_ and transaminase elevation slopes in longitudinal analysis. Given that weight-based dosing was used in the HIRIF trial and that ethambutol exposure correlated with pyrazinamide exposure, the strongest predictor of hepatotoxicity (Supplementary Figure 1), this association does not necessarily imply that ethambutol exposure itself is a direct cause of hepatotoxicity. Rather, it may reflect the influence of weight-based dosing strategies and the interplay between pyrazinamide and ethambutol exposures. One case series reported a high incidence of hepatotoxicity (58%) with combined ethambutol and pyrazinamide therapy for latent TB infection [38], but the independent hepatotoxic potential of ethambutol remains unclear due to the complex nature of multidrug treatment involving pyrazinamide co-administration. Ethambutol has been linked to hepatotoxicity in only a few case reports. One described cholestatic jaundice occurring two months after initiation of ethambutol, isoniazid, and streptomycin, and recurring upon rechallenge with ethambutol alone [39]. This provided convincing evidence of ethambutol’s role, although the cholestatic pattern is atypical in anti-TB drug-induced hepatotoxicity.

In conclusion, higher exposures to pyrazinamide and isoniazid, and slow *NAT2* acetylator status, were each associated with increased hepatotoxicity, and only pyrazinamide exposure was associated when considering pharmacokinetic variables together. Rifampin exposure as achieved by doses of up to 20 mg/kg/day was not associated with hepatotoxicity. Because our analysis focused specifically on hepatotoxicity, one of the most common adverse events in the standard regimen for DS-TB, it does not necessarily reflect overall tolerability. Nevertheless, our findings support further evaluation of rifampin at doses up to 20 mg/kg/day in a larger clinical trial and its potential clinical use from a safety perspective, providing valuable insights into the hepatotoxicity risk associated with drugs within this regimen.

## Supplementary Material

Online supplement

## Data Availability:

The data are available upon request.

## Figures and Tables

**Figure 1.**
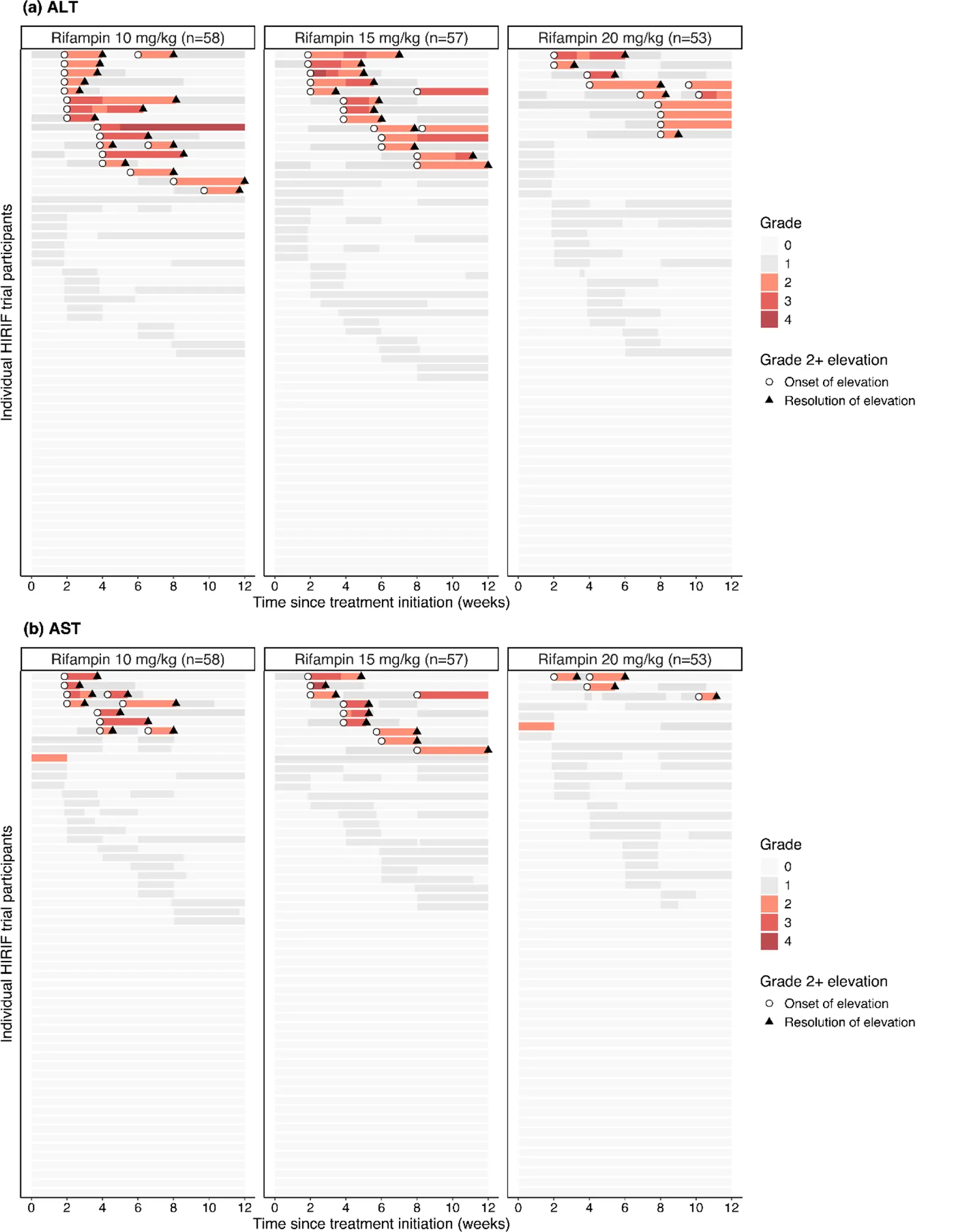
Longitudinal profiles of (a) ALT grade and (b) AST grade over time since treatment initiation for individual participants, stratified by randomized rifampin dose. The bar colors represent severity grade of ALT or AST elevation. The open circle and filled triangle represent the onset and resolution of grade 2 or higher elevation, respectively. Abbreviations: ALT, alanine transaminase; AST, aspartate transaminase.

**Figure 2.**
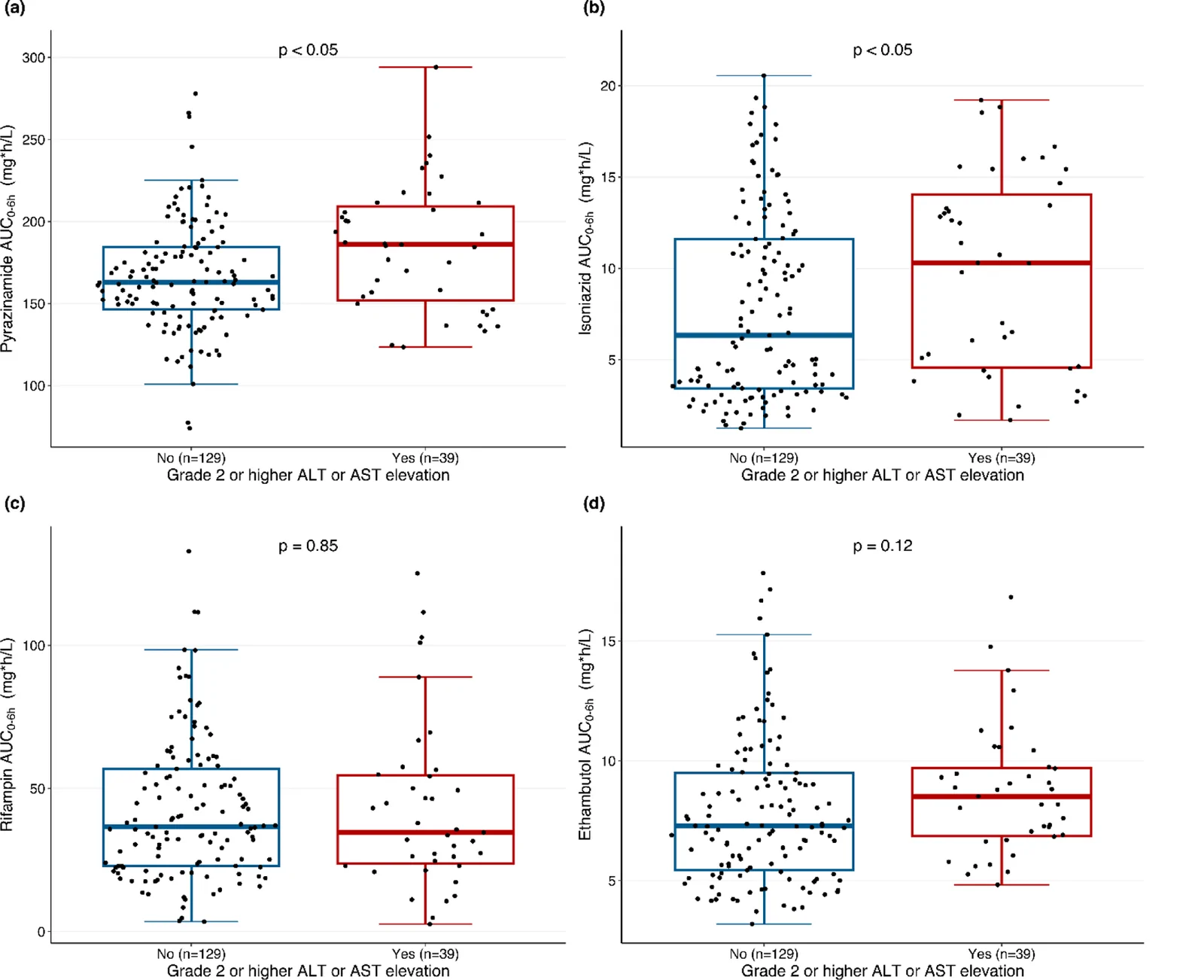
Comparison of (a) pyrazinamide, (b) isoniazid, (c) rifampin, and (d) ethambutol AUC_0–6h_ between participants with and without grade 2 or higher ALT or AST elevation. The filled circles represent the observed values. The lower and upper hinges represent the 25th and 75th percentiles, and the middle line represents the median. The whiskers extend from the hinge to the smallest or largest value no further than 1.5 times the interquartile range from the hinge. Significance was evaluated using an independent two-sample *t*-test. Abbreviations: ALT, alanine transaminase; AST, aspartate transaminase; AUC_0–6h_, area under the concentration-time curve from 0 to 6 hours.

**Figure 3.**
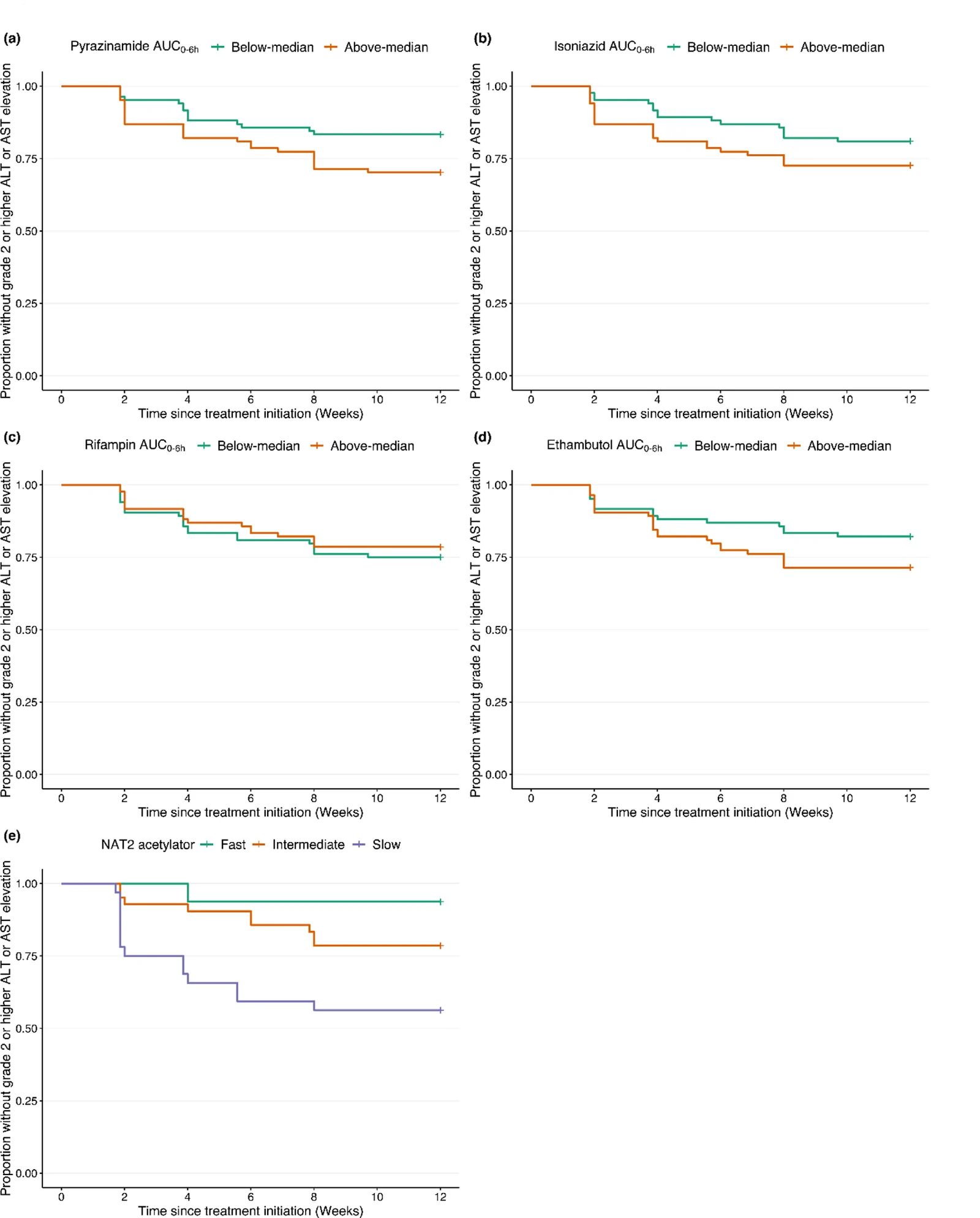
Kaplan-Meier curves for first grade 2 or higher ALT or AST elevation, stratified by median exposure to (a) pyrazinamide, (b) isoniazid, (c) rifampin, and (d) ethambutol as defined by AUC_0–6h_, and by (e) *NAT2* acetylator status. Abbreviations: ALT, alanine transaminase; AST, aspartate transaminase; AUC_0–6h_, area under the concentration-time curve from 0 to 6 hours; *NAT2*, N-acetyltransferase 2.

**Figure 4.**
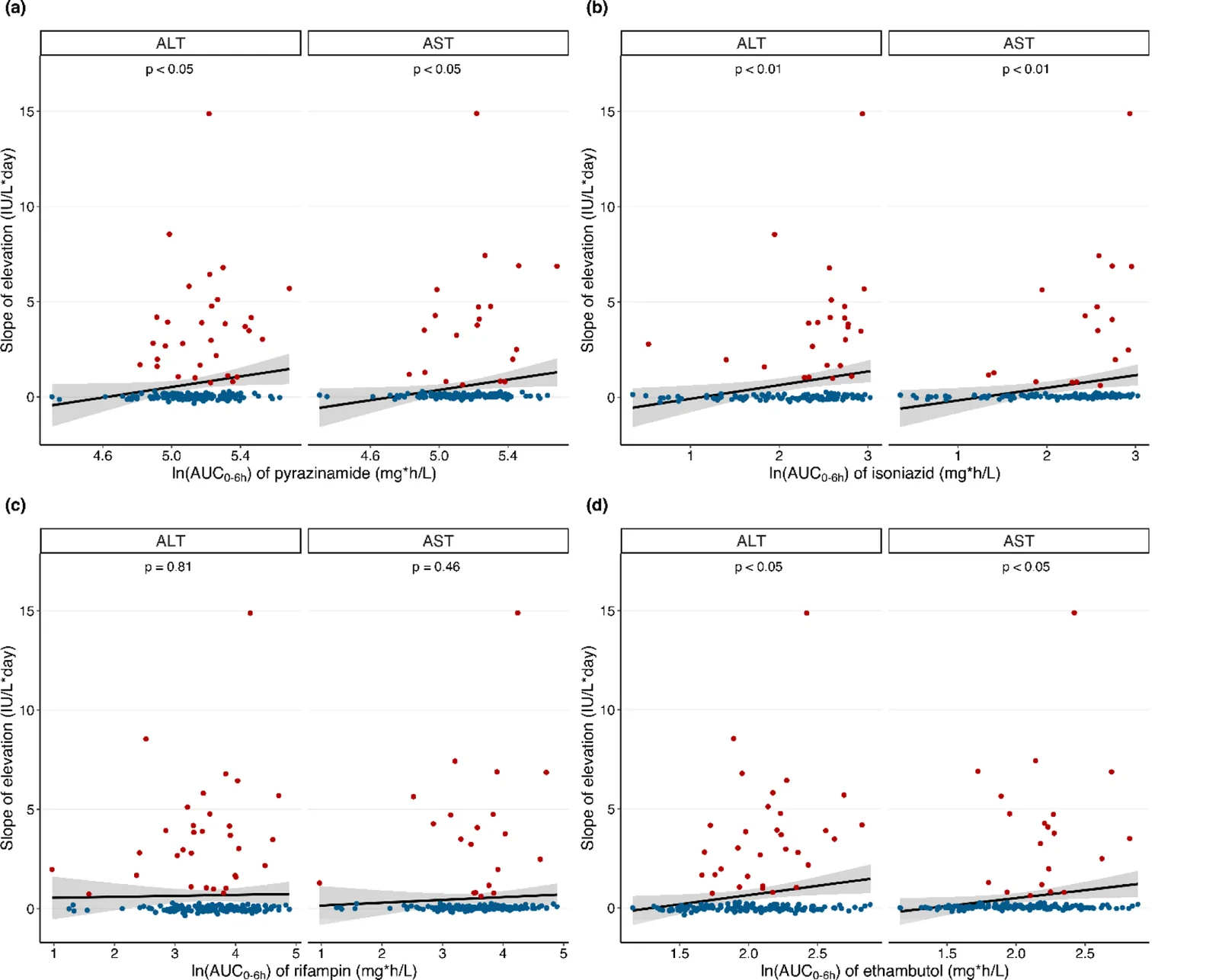
Association between observed (a) pyrazinamide, (b) isoniazid, (c) rifampin, and (d) ethambutol AUC_0–6h_ and the longitudinal model-predicted slope of ALT and AST elevation. The blue and red filled circles represent the observed values in the model-assigned low and high ALT and AST elevation groups, respectively. The lines represent the predicted mean lines from linear regression, and the shaded areas represent the 95% confidence intervals for the regression lines. Significance was evaluated using linear regression. Abbreviations: ALT, alanine transaminase; AST, aspartate transaminase; AUC_0–6h_, area under the concentration-time curve from 0 to 6 hours.

**Table 1.** Characteristics of HIRIF trial participants by randomized rifampin dose group.

	Randomized Rifampin Dose			
	10 mg/kg (n=58)	15 mg/kg (n=57)	20 mg/kg (n=53)	Total (n=168)

***Baseline characteristics***				
Age (years)	24 (18 – 60)	25 (18 – 59)	26 (18 – 59)	25 (18 – 60)
Weight (kg)	53.3 (36.1 – 90.5)	55.2 (36.4 – 88.5)	55.5 (36.0 – 88.2)	54.0 (36.0 – 90.5)
Body mass index (kg/m^2^)	21.2 (15.7 – 34.9)	21.2 (15.9 – 29.4)	21.4 (15.4 – 36.7)	21.2 (15.4 – 36.7)
Sex				
Male	37 (63.8%)	39 (68.4%)	31 (58.5%)	107 (63.7%)
Female	21 (36.2%)	18 (31.6%)	22 (41.5%)	61 (36.3%)
Baseline ALT (IU/L)	24 (6 – 79)	20 (7 – 63)	22 (6 – 59)	22 (6 – 79)
Baseline AST (IU/L)	22 (12 – 77)	20 (12 – 64)	19 (11 – 85)	20 (11 – 85)
Rifampin dose (mg)	450 (300 – 750)	900 (450 – 1,200)	1,200 (600 – 1,500)	600 (300 – 1,500)

***Pharmacokinetics***				
Pyrazinamide AUC_0-6h_ (h∙mg/L)	167.8 (74.1 – 277.9)	164.4 (114.7 – 294.0)	163.9 (77.4 – 266.2)	166.3 (74.1 – 294.0)
Pyrazinamide C_max_ (mg/L)	35.2 (21.1 – 66.4)	33.4 (19.6 – 61.9)	34.4 (18.9 – 54.3)	34.6 (18.9 – 66.4)
Isoniazid AUC_0-6h_ (h∙mg/L)	5.6 (1.4 – 18.8)	8.2 (1.6 – 20.6)	6.5 (1.3 – 19.3)	6.9 (1.3 – 20.6)
Isoniazid C_max_ (mg/L)	2.4 (0.5 – 6.3)	2.5 (0.5 – 6.0)	2.2 (0.6 – 6.7)	2.3 (0.5 – 6.7)
Rifampin AUC_0-6h_ (h∙mg/L)	24.9 (2.6 – 57.9)	43.1 (3.5 – 111.6)	55.5 (13.6 – 132.9)	36.2 (2.6 – 132.9)
Rifampin C_max_ (mg/L)	6.2 (0.6 – 12.5)	10.2 (0.6 – 27)	13.3 (3.9 – 39.2)	9.2 (0.6 – 39.2)
Ethambutol AUC_0-6h_ (h∙mg/L)	7.2 (3.7 – 17.2)	7.9 (3.8 – 16.8)	7.8 (3.2 – 17.8)	7.4 (3.2 – 17.8)
Ethambutol C_max_ (mg/L)	1.6 (0.7 – 5.2)	2.0 (0.7 – 4.5)	1.8 (0.9 – 5.6)	1.7 (0.7 – 5.6)

Data are expressed as median (range) except for sex which is expressed as number of participants (%).

Abbreviations: ALT, alanine transaminase; AST, aspartate transaminase; AUC_0-6h_, area under the concentration-time curve from 0 to 6 hours; C_max_, maximum plasma concentration.

**Table 2.** Cox proportional hazard ratios for grade 2 or higher ALT or AST elevation in univariable, full, and final models.

Variable	Participant (n)	Event (n)	Univariable model	Full model^a^	Final model

			HR (95% CI)	HR (95% CI)	HR (95% CI)

Pyrazinamide AUC_0-6h_ (per 50 h∙mg/L increase)	168	39	1.85 (1.23 – 2.81)**	1.76 (0.99 – 3.15)	1.85 (1.23 – 2.81)**
Pyrazinamide C_max_ (per 10 mg/L increase)	168	39	1.56 (1.10 – 2.22)*	-	-
Isoniazid AUC_0-6h_ (per 5 h∙mg/L increase)	168	39	1.40 (1.04 – 1.87)*	1.35 (0.97 – 1.87)	-
Isoniazid C_max_ (per 1 mg/L increase)	168	39	1.29 (1.03 – 1.61)*	-	-
Rifampin AUC_0-6h_ (per 50 h∙mg/L increase)	168	39	1.07 (0.58 – 1.96)	0.63 (0.31 – 1.29)	-
Rifampin C_max_ (per 10 mg/L increase)	168	39	0.97 (0.58 – 1.63)	-	-
Ethambutol AUC_0-6h_ (per 5 h∙mg/L increase)	168	39	1.46 (0.91 – 2.34)	1.52 (0.88 – 2.63)	-
Ethambutol C_max_ (per 1 mg/L increase)	168	39	1.15 (0.84 – 1.57)	-	-
Rifampin dose (per 150 mg increase)	180	46	1.05 (0.91 – 1.22)	-	-
Rifampin 15 mg/kg/day (relative to 10 mg/kg/day)	60 (vs. 60)	16 (vs. 16)	0.99 (0.49 – 1.97)	-	-
Rifampin 20 mg/kg/day (relative to 10 mg/kg/day)	60 (vs. 60)	14 (vs. 16)	0.84 (0.41 – 1.71)	-	-
*NAT2* intermediate acetylator (relative to *NAT2* fast acetylator)	42 (vs. 16)	9 (vs. 1)	3.63 (0.46 – 28.62)	-	-
					
*NAT2* slow acetylator (relative to *NAT2* fast acetylator)	32 (vs. 16)	14 (vs. 1)	9.32 (1.22 – 70.92)*	-	-
*NAT2* slow acetylator (relative to *NAT2* intermediate acetylator)	32 (vs. 42)	14 (vs. 9)	2.57 (1.11 – 5.94)*	-	-

a The full multivariable model includes all prespecified predictors of interest (excluding C_max_ parameters and *NAT2* acetylator status) and potential confounders, regardless of statistical significance.

* p < 0.05;

** p < 0.01

Abbreviations: ALT, alanine transaminase; AST, aspartate transaminase; AUC_0-6h_, area under the concentration-time curve from 0 to 6 hours; CI, confidence interval; C_max_, maximum plasma concentration; HR, hazard ratio; *NAT2*, N-acetyltransferase 2.
