# Supplementary material for: Pharmacokinetic and pharmacogenomic predictors of hepatotoxicity in the HIRIF trial for drug-susceptible tuberculosis
Source: Clin Infect Dis. Author manuscript; Available in PMC 2026 Oct 8. (PMC13647239; doi:10.1093/cid/ciag141)
Supplement: Supplementary file 1 [file NIHMS2161057-supplement-Online_supplement.docx]

**Supplementary Materials**

**Pharmacokinetic and pharmacogenomic predictors of hepatotoxicity in the HIRIF trial
for drug-susceptible tuberculosis**

**Authors**

Eunsol Yang^1^, Pieter Van Brantegem^1^, Charles A. Peloquin^2^, Meredith B. Brooks^3^, Julia M. Coit^4^, Dante Vargas Vásquez^5^, Epifanio Sánchez Garavito^6^, Roger I. Calderón^7,8^, Judith Jiménez^9^, Karen Tintaya^9^, Dalicxa García^9^, Elna Osso^4^, Elizabeth A. Mackay^10^, Gareth Platt^10^, Leonid Lecca^4,9^, Radojka M. Savic^1,11^, Geraint R. Davies^10^, Carole D. Mitnick^4,12,13,^*, Gustavo E. Velásquez^11,14,^*

** = Contributed equally*

**Affiliations**

^1^ Department of Bioengineering and Therapeutic Sciences, University of California, San Francisco, San Francisco, California, USA

^2^ College of Pharmacy and Emerging Pathogens Institute, University of Florida, Gainesville, Florida, USA

^3^ Department of Global Health, Boston University School of Public Health, Boston, Massachusetts, USA

^4^ Department of Global Health and Social Medicine, Harvard Medical School, Boston, Massachusetts, USA

^5^ Hospital Nacional Hipólito Unanue, Lima, Peru

^6^ Hospital Nacional Sergio Bernales, Lima, Peru

^7^ Advanced Research and Health, Lima, Peru

^8^ Facultad de Medicina Humana, Universidad de San Martín de Porres, Lima, Peru

^9^ Socios En Salud Sucursal Peru, Lima, Peru

^10^ Department of Clinical Infection, Microbiology, and Immunology, University of Liverpool, Liverpool, United Kingdom

^11^ UCSF Center for Tuberculosis, Institute for Global Health Sciences, University of California, San Francisco, San Francisco, California, USA

^12^ Division of Global Health Equity, Brigham and Women's Hospital, Boston, Massachusetts, USA

^13^ Partners In Health, Boston, Massachusetts, USA

^14^ Division of HIV, Infectious Diseases, and Global Medicine, University of California, San Francisco, San Francisco, California, USA

**Corresponding author:**

Gustavo E. Velásquez, MD, MPH

Division of HIV, Infectious Diseases, and Global Medicine

University of California, San Francisco

2540 23rd Street, San Francisco, CA 94110, USA

Email: gustavo.velasquez@ucsf.edu

**Alternate corresponding author:**

Carole D. Mitnick, ScD

Department of Global Health and Social Medicine

Harvard Medical School

641 Huntington Avenue, Boston, MA 02115, USA

Email: carole_mitnick@hms.harvard.edu

**Table of Contents**

[**Supplementary Figure 1.** Correlation of AUC_0-6h_ between antituberculosis drugs.	3](#_Toc217131753)

[**Supplementary Figure 2.** Box-and-whisker plot of isoniazid AUC_0-6h_ by *NAT2* acetylator status.	4](#_Toc217131754)

[**Supplementary Figure 3.** Longitudinal (a) ALT and (b) AST profiles over time.	5](#_Toc217131755)

[**Supplementary Figure 4.** Longitudinal (a) ALT and (b) AST profiles over time, stratified by model-assigned mixture groups for low and high ALT or AST elevation.	6](#_Toc217131756)

[**Supplementary Figure 5.** Visual predictive check of longitudinal (a) ALT and (b) AST models.	7](#_Toc217131757)

[**Supplementary Figure 6.** Comparison of observed (a) pyrazinamide, (b) isoniazid, (c) rifampin, and (d) ethambutol AUC_0-6h_ between the model-assigned ALT and AST elevation groups.	8](#_Toc217131758)

[**Supplementary Table 1.** Cox proportional hazard ratios for grade 2 or higher ALT or AST elevation in univariable models.	9](#_Toc217131771)

[**Supplementary Table 2.** Cox proportional hazard ratios for grade 2 or higher ALT or AST elevation in models adjusted by randomized rifampin dose.	10](#_Toc217131772)

[**Supplementary Table 3.** Cox proportional hazard ratios for grade 2 or higher ALT or AST elevation in univariable and full models in subset with available pharmacokinetic and *NAT2* genotype data.	11](#_Toc217131773)

[**Supplementary Table 4.** Parameter estimates of longitudinal ALT and AST models.	12](#_Toc217131774)

**Supplementary Figures**

**Supplementary Figure 1.** Correlation of AUC_0-6h_ between antituberculosis drugs. The filled circles represent the observed values, and colors (top row) represent randomized rifampin dose. The lines represent the predicted values from linear regression, and the shaded areas represent the 95% confidence intervals for the predicted values. Significance was evaluated using linear regression. Abbreviations: AUC_0-6h_, area under the concentration-time curve from 0 to 6 hours; ρ, Pearson correlation coefficient.

**Supplementary Figure 2.** Box-and-whisker plot of isoniazid AUC_0-6h_ by *NAT2* acetylator status. The filled circles represent the observed values. The lower and upper hinges represent the 25th and 75th percentiles, and the middle line represents the median. The whiskers extend from the hinge to the smallest or largest value no further than 1.5 times the interquartile range from the hinge. Abbreviations: AUC_0-6h_, area under the concentration-time curve from 0 to 6 hours; *NAT2*, N-acetyltransferase 2.

**Supplementary Figure 3.** Longitudinal (a) ALT and (b) AST profiles over time. The black solid lines individual participant represent ALT and AST trajectories up to the first grade 2 or higher elevation. The black dashed lines represent individual participant ALT and AST trajectories after the first grade 2 or higher elevation. The horizontal lines (upper red dashed line and lower red dash-dotted line) represent the cutoff values for grade 2 ALT or AST elevations for males and females, respectively. Abbreviations: ALT, alanine transaminase; AST, aspartate transaminase.

**Supplementary Figure 4.** Longitudinal (a) ALT and (b) AST profiles over time, stratified by model-assigned mixture groups for low and high ALT or AST elevation. The ALT and AST levels for individual participants were censored at the first grade 2 or higher elevation, respectively. Abbreviations: ALT, alanine transaminase; AST, aspartate transaminase.

**Supplementary Figure 5.** Visual predictive check of longitudinal (a) ALT and (b) AST models. The observed ALT and AST data were truncated at 200 IU/L in the plot for better visibility; all data were included in the visual predictive check analyses. The open circles represent the observed data. The solid lines represent the medians of the observed data. The dashed lines represent the 10^th^ and 90^th^ percentiles of the observed data. The shaded areas represent the 95% confidence intervals for the medians and for the 10^th^ and 90^th^ percentiles of the predicted data. Abbreviations: ALT, alanine transaminase; AST, aspartate transaminase.

**Supplementary Figure 6.** Comparison of observed (a) pyrazinamide, (b) isoniazid, (c) rifampin, and (d) ethambutol AUC_0-6h_ between the model-assigned ALT and AST elevation groups. The filled circles represent the observed values. The lower and upper hinges represent the 25th and 75th percentiles, and the middle line represents the median. The whiskers extend from the hinge to the smallest or largest value no further than 1.5 times the interquartile range from the hinge. Significance was evaluated using an independent two-sample t-test. Abbreviations: ALT, alanine transaminase; AST, aspartate transaminase; AUC_0-6h_, area under the concentration-time curve from 0 to 6 hours.
**Supplementary Tables**

**Supplementary Table 1.** Cox proportional hazard ratios for grade 2 or higher ALT or AST elevation in univariable models.

|  |  |  | **Univariable model** |
| --- | --- | --- | --- |
| **Variable** | **Participant (n)** | **Event (n)** | **HR (95% CI)** |
| Age  (per 10 years increase) | 168 | 39 | 1.17 (0.92 – 1.50) |
| Female  (relative to male) | 168 | 39 | 1.13 (0.59 – 2.15) |
| Weight^a^  (per 10 kg increase) | 168 | 39 | 1.14 (0.84 – 1.56) |
| Body mass index  (per 1 kg/m^2^ increase) | 168 | 39 | 1.04 (0.96 – 1.14) |
| Baseline ALT  (per 10 IU/L increase) | 168 | 39 | 1.08 (0.89 – 1.31) |
| Baseline AST  (per 10 IU/L increase) | 168 | 39 | 0.96 (0.73 – 1.26) |
| Baseline ALP  (per 10 IU/L increase) | 168 | 39 | 0.97 (0.91 – 1.04) |
| Baseline total bilirubin  (per 1 mg/dL increase) | 168 | 39 | 1.33 (0.40 – 4.43) |

* p < 0.05

^a^ The proportional hazards assumption was not met.

Abbreviations: ALP, alkaline phosphatase; ALT, alanine transaminase; AST, aspartate transaminase; CI, confidence interval; HR, hazard ratio.

**Supplementary Table 2.** Cox proportional hazard ratios for grade 2 or higher ALT or AST elevation in models adjusted by randomized rifampin dose.

|  |  |  | **Adjusted model** |
| --- | --- | --- | --- |
| **Predictors** | **Participant (n)** | **Event (n)** | **HR (95% CI)** |
| Pyrazinamide AUC_0-6h_  (per 50 h∙mg/L increase) | 168 | 39 | 1.88 (1.23 – 2.87)** |
| Pyrazinamide C_max_  (per 10 mg/L increase) | 168 | 39 | 1.55 (1.09 – 2.20)* |
| Isoniazid AUC_0-6h_  (per 5 h∙mg/L increase) | 168 | 39 | 1.43 (1.06 – 1.92)* |
| Isoniazid C_max_  (per 1 mg/L increase) | 168 | 39 | 1.30 (1.04 – 1.62)* |
| Rifampin AUC_0-6h_  (per 50 h∙mg/L increase) | 168 | 39 | 1.50 (0.74 – 3.06) |
| Rifampin C_max_  (per 10 mg/L increase) | 168 | 39 | 1.24 (0.68 – 2.23) |
| Ethambutol AUC_0-6h_  (per every 5 h∙mg/L increase) | 168 | 39 | 1.44 (0.90 – 2.3) |
| Ethambutol C_max_  (per 1 mg/L increase) | 168 | 39 | 1.14 (0.84 – 1.56) |
| *NAT2* intermediate acetylator  (relative to NAT2 fast acetylator) | 42  (vs. 16) | 9  (vs. 1) | 3.63 (0.46 – 28.75) |
| *NAT2* slow acetylator  (relative to NAT2 fast acetylator) | 32  (vs. 16) | 14  (vs. 1) | 9.27 (1.22 – 70.8)* |
| *NAT2* slow acetylator  (relative to NAT2 intermediate acetylator) | 32  (vs. 42) | 14  (vs. 9) | 2.57 (1.11 – 5.94)* |

* p < 0.05; ** p < 0.01

Abbreviations: ALT, alanine transaminase; AST, aspartate transaminase; AUC_0-6h_, area under the concentration-time curve from 0 to 6 hours; CI, confidence interval; C_max_, maximum plasma concentration; HR, hazard ratio; *NAT2*, N-acetyltransferase 2.

**Supplementary Table 3.** Cox proportional hazard ratios for grade 2 or higher ALT or AST elevation in univariable and full models in subset with available pharmacokinetic and *NAT2* genotype data.

|  |  |  | **Univariable model** | **Full model^a^** |
| --- | --- | --- | --- | --- |
| **Predictors** | **Participant (n)** | **Event (n)** | **HR (95% CI)** | **HR (95% CI)** |
| Isoniazid AUC_0-6h_  (per 5 h∙mg/L increase) | 84 | 20 | 1.54 (1.05 – 2.28)* | 1.06 (0.53 – 2.12) |
| *NAT2* intermediate acetylator  (relative to NAT2 fast acetylator) | 40  (vs. 15) | 8  (vs. 1) | 3.13 (0.39 – 25.00) | 3.04 (0.37 – 25.07) |
| *NAT2* slow acetylator  (relative to NAT2 fast acetylator) | 29  (vs. 15) | 11  (vs. 1) | 7.16 (0.92 – 55.51) | 6.33 (0.48 – 82.97) |

^a^ The full multivariable model includes isoniazid AUC_0-6h_ and *NAT2* acetylator status, regardless of statistical significance.

* p < 0.05; ** p < 0.01

Abbreviations: ALT, alanine transaminase; AST, aspartate transaminase; AUC_0-6h_, area under the concentration-time curve from 0 to 6 hours; CI, confidence interval; HR, hazard ratio; *NAT2*, N-acetyltransferase 2.

**Supplementary Table 4.** Parameter estimates of longitudinal ALT and AST models.

| **Parameters** | **ALT model** | **AST model** |
| --- | --- | --- |
|  | **Estimates (RSE%)** | **Estimates (RSE%)** |
| Baseline (IU/L) | 25.0 (3.8) | 23.3 (3.5) |
| Slope, low elevation (IU/L*day) | 0.021 (98.6) | 0.0716 (20.5) |
| Slope, high elevation (IU/L*day) | 3.49 (33.8) | 3.91 (40.9) |
| % Participants with high elevation | 19.9 (4.9) | 12.5 (3.5) |
| IIV Baseline (CV%) | 39.2 (17.8) | 30.2 (17.2) |
| IIV Slope, low elevation (CV%) | 733.1 (29.7) | 157.4 (26.3) |
| IIV Slope, high elevation (CV%) | 91.5 (26.8) | 92.2 (35.9) |
| Proportional residual error (%) | 32.6 (2.6) | 26.3 (2.4) |

Abbreviations: ALT, alanine transaminase; AST, aspartate transaminase; CV, coefficient of variation; IIV, inter-individual variability; RSE, relative standard error.
